# Kuru memories from 1957

**DOI:** 10.1098/rstb.2008.4028

**Published:** 2008-11-27

**Authors:** Roy F.R. Scragg

**Affiliations:** 36 South EsplanadeGlenelg, SA 5045, Australia

The historic letter written by Vin Zigas to John Gunther on Christmas Day 1956 generated a quick reply as follows.

7th January 1957

GUNTHER to ZIGAS

Dear Dr Zigas

Thank you for your extremely interesting report. I hope you will concentrate every effort on eradicating the disease. I am going to ask Dr Anderson to help you. I am asking Anderson if he thinks he should visit the area with you.

It seems too early yet for any comment from me, but the restricted area of distribution would suggest an arthropod vector.

Yours sincerely

J. T. Gunther

GUNTHER to ANDERSON

Dear Dr Anderson

I have just received a letter from Dr Zigas describing an epidemic of an encephalitic-like disease at Okapa.

He informs me he sent a brain and blood samples to you. Before I make an approach to Sir Mac. through the Minister of Territories, do you think you should visit the area?

Yours faithfully

J. T. Gunther

These and other letters, including a letter from Dr L. I. Taft reporting on the first brain the Walter and Eliza Hall Institute (WEHI) received, are not included in the published Gajdusek correspondence of the early days.

On 4th February, John Gunther sent another letter to Gray Anderson in which he wrote, ‘The Government Anthropologist has visited Fore and informs me the Administration is losing prestige because of its inability to meet the sorcery, Kuru. He further gave me a description of the disease which appears to be much like that presented by Dr Zigas. One can only suggest that it can be described as Encephalitis Lethargica, that it is endemic in the regions, and that its epidemiology requires further investigation. I am wondering whether this would be in your line of work and whether you would be interested in undertaking the investigation.’

Anderson replied on 12th February:

The Walter and Eliza Hall Institute of Medical Research

C/o Royal Melbourne

Hospital Post Office,

Melbourne,

Victoria, Australia

12th February 1957.

Dr J. T. Gunther,

Director of Public Health,

Port Moresby,

Papua.

In reply to your letter of 32/35 M.215 of 4th February 1957.

Dear Doctor Gunther

I would be interested to investigate the Encephalitis at Okapa. I assume from your earlier letter (32/35) (M.2243) that you wish first to make a formal approach to Sir Macfarlane through the Minister of Territories.

Both my wife and Sir Macfarlane have independently expressed concern at the possible danger of investigating ‘sorcery’ among the Fore people. I assume that this area is fully controlled and that there would be no appreciable risk from hostile natives singly or en masse. I would like to convey to my wife and Sir Mac your personal assurance on this point before visiting the area.

I have discussed this outbreak with Professor F. Shaw, Department of Pharmacology of the University of Melbourne. He agrees that certain plant toxins might produce a similar clinical condition though rarely pyrexia. He is making further enquiries.

I enclose some suggestions about possible further steps to investigate the outbreak. I have written to Doctor Zigas asking for material mentioned in paragraph I of this enclosure.

If cases at Okapa occur in epidemics it might be best to visit the area just at the beginning of an epidemic. If cases occur throughout the year my personal commitments would allow me to visit the area at short notice and at a time convenient to you.

I shall wait to hear from you.

Yours sincerely,

S. G. Anderson.

Enc.

(Attachment to letter from Anderson to Gunther, 12 February 1957)

## Possible further steps to investigate the outbreak at Okapa

- Could we obtain more material and information about the cases at Okapa?
  - Clinical history of the native whose brain was sent to us, particularly including date of onset.
  - Typical temperature charts of cases or general description of temperature–onset magnitude and duration.
  - An estimate of seasonal (monthly) incidence of onsets of cases.
  - As many sera as possible taken from cases at least six weeks after onset (to eliminate certain of the Rickettsiae). Each serum to be 4 ml, not haemolysed.
  - The following specimens from cases taken as early as possible after onset:
    - CSF cell count done at Okapa.
    - CSF sent to Melbourne or local laboratory for biochemistry.
    - Faeces in normal saline with 100 units of penicillin per ml refrigerated and sent to us airmail in sealed container.
    - Heparinized blood 10 c.c. refrigerated and sent to us airmail.
    - Pieces half an inch cube from right cortex, right basal ganglia, right medulla and right dorsal cord in glycerol saline for virus isolation at Hall Institute.
    - The remainder of each brain and cord into 10% formalin in saline. The brain fixes better if it is cut in slices 1 inch thick.
    - Formalized pieces of other organs from same cases–e.g. muscle of affected areas, liver, heart, lung, kidney, spleen, etc.
- A visit to the Okapa area would allow detailed discussion of the habits, customs and food sources of the people and their contact with animals and ectoparasites, both in the area affected and in surrounding ‘clean’ areas. Would the local terrain allow jeep transport? If there is a seasonal epidemic of cases, this visit might best be made concurrently with the beginning of the epidemic.
- If I were to visit Okapa (with Dr Zigas), I would press for the services of an A.D.O. well supervised in the region and adept at handling the natives and gaining their confidence. An anthropologist might be most valuable also.
- It might become desirable to have a concurrent survey of the local flora by a botanist interested in the pharmacology of plants.
- As a result of the above two approaches, it might be possible to collect specimens either of toxic plants or of hosts and vectors of the presumed infectious agent. Such specimens might best be collected just prior to and concurrently with a seasonal epidemic of cases and these specimens could probably be examined in Melbourne.

On 15th February, Gunther wrote to Burnet saying that he would visit Kainantu on 18th February. On the same day, he minuted the file to me to see him and discussed his disappointment at having to go to Canberra and asked me to visit for him. My report on the three cases I saw is part of the history of kuru and was written by a physician who had just spent three months walking the medical wards as a postgraduate student.

I was fascinated by kuru and the finding of a new condition in a country full of many well-known tropical and introduced diseases. On 25th February, Gunther told me of his promotion to Assistant Administrator and on 4th March, I became the Director of Public Health.

On 7th March, I met Carleton for the first time. He had been in New Britain in mid-1956 while I was on leave and Clarissa de Derka had mentioned his planned visit. As one young physician to another, I disgorged my fascination with kuru and Carleton quickly caught the contagion. I gave him access to the file containing all the letters and discussed the geography of Lufa, where he planned to visit Ian Burnet, son of Sir Macfarlane, and its proximity to Okapa. I mentioned that if he had time he might visit Dr Zigas and add his insight to the condition.

One could liken his reaction to showing a red flag to a bull. He arrived at Goroka on 11th March and obtained directions for Kainantu from Dr Symes, the Regional Medical Officer, and was there that day.

On 13th March, he wrote a long letter to Sir Macfarlane Burnet, Ian Wood and S. Gray Anderson at WEHI explaining his presence in the area studying the condition that Anderson was to have studied, and early in the letter states:

The more I read of the literature and correspondence, the more obvious was it that I had no intention of stepping into your project, but likewise I was somewhat disappointed that I had heard no word about kuru and its interesting and intriguing problems.

The Institute doctors did not ever respond in writing to the last line. It was not clear to me at the time of the recent London meeting whether Carleton knew of kuru before he came to New Guinea. At the conference, I said, ‘There was a culture of secrecy at the Hall surrounding kuru and Gajdusek was kept out of the link.’

Since the meeting, I have had my first opportunity to discuss this issue with Dr Gray Anderson. He clearly remembers a meeting with Burnet and Gajdusek discussing a neurological problem but is not sure whether it was kuru or not. Since returning home, I have reread the early letters including those about the many specimens sent to the Hall Institute and the discussion of the possible visit of Anderson with the Board. Against the scientific culture of the Hall where everyone was encouraged to discuss their research with other scientists at tea time, there is now no question in my mind that Carleton knew of kuru before he saw me in Moresby on 7th March. I think he was frustrated by the three-month delay from the first specimens reaching the Hall with no sign of a scientific visit to help sort out a killing disease.

### The professors

In late October 1957, Carleton and his party set out from Okapa to traverse Papua down the Lamari River into the Gulf of Papua and to Port Moresby to get help to facilitate the clearance of all the blocks in the research programme. He arrived in Moresby on Saturday 1st November.

That same night, my wife and I were hosting a party for six visiting professors and deans from Australian universities who had been invited to attend the new Port Moresby General Hospital opening on the Sunday. Carleton was invited to meet them. He arrived as he had travelled in shirt and shorts for the dinner party with only one sandshoe as the other had been lost on the trip. The professors were spellbound as they heard first hand his description of the clinical, anthropological and environmental aspects of kuru.

They were like bees around a honey pot trying to understand what they saw to be a new disease being described to them for the first time. Their interest generated questions on many aspects and suggestions of how they might be able to assist with personnel and laboratory support. Profs Sydney Sunderland, a neuroanatomist from Melbourne, John Eccles, a neurophysiologist from Canberra, and Norrie Robson, a physician from Adelaide, were the most interested and proceeded in the next week to visit Kainantu and Okapa.

Prof. Robson was the one who generated interest in his medical school, which led to the subsequent involvement with kuru of Henry Bennett, Bronte Gabb, John Cleland, Donald Simpson, Harry Lander, Michael Alpers and others.

